# Malaria and an Amoebic Abscess in a Returning Traveler from Liberia

**DOI:** 10.1155/2023/1466397

**Published:** 2023-07-20

**Authors:** Mhd Mustafa Albitar, Nafiza Martini, Sandy Alkhalil, Tamim Alsuliman, Ali Alrstom

**Affiliations:** ^1^Damascus University, Faculty of Medicine, Damascus, Syria; ^2^Stemosis for Scientific Research, Damascus, Syria; ^3^Hematology and Cell Therapy Department, Saint-Antoine Hospital, AP-HP Sorbonne University, Paris, France; ^4^Infectious Disease, Internal Medicine Department, Al-Mouwasat (Damascus University Affiliated) Hospital, Damascus, Syria

## Abstract

Malaria is an infectious *Anopheles* mosquito-borne disease caused by five different eukaryotic protozoa parasites. Amoebiasis is a parasitic infection caused by *Entamoeba histolytica*. Both diseases are widespread in Liberia. A returning traveler was diagnosed and treated for malaria, and 20 days later, an amoebic liver abscess was discovered, meaning that the malaria infection masked the amoebic infection, which emphasizes the importance of a complete examination of returning travelers, especially for those returning from Sub-Saharan Africa, where coinfections are more common. Herein, we propose that the relationship between Malaria and amoebic liver abscesses should be explored by researching the effects of malaria on ferritin levels and the immune components in the liver and whether it helps the emergence of hepatic amoebic abscesses or not.

## 1. Introduction and Importance

Malaria is an infectious *Anopheles *mosquito-borne disease caused by five different eukaryotic protozoa parasites of the genus Plasmodia, one of which is *Plasmodium falciparum*, and it is responsible for 90% of global deaths from malaria [1].

The infection is usually associated with a travel history to infected countries, of which is Liberia, where the entire population is at high risk of catching the disease [2]. Malaria symptoms are commonly nonspecific; thus, clinical diagnosis is difficult [1].

On the other hand, amoebiasis is a parasitic infection caused by *Entamoeba histolytica* and is transmitted via the fecal-oral route. The infection varies in severity from asymptomatic cases to complicated ones that may include liver abscesses [3].

Amoebiasis remains a global problem that is especially present in developing countries such as Syria and Liberia, where it mainly spreads through contaminated water supplies [3] Around 50 million people contract amoebiasis worldwide, with deaths as high as 100,000 deaths annually [3], making it the second most mortal parasitic disease [4].

Like malaria, amoebiasis is commonly associated with a travel history to infected regions including Liberia. Most patients who develop liver abscesses also develop fever, right hypochondriac pain, and digestive symptoms. The symptoms usually manifest 2–4 weeks after infection [3].

Travelers to infected regions are advised to follow precautionary measures. For preventing malaria, protection from the mosquito bite is usually achieved by applying insect repellents on naked skin such as diethyltoluamide (DEET) and using insecticide-treated nets or clothing [5]. Infection prevention is essential, particularly with the rising concerns of artemisinin-based combination therapy resistance, which will require further research into new antimalarial drugs, especially starting from medicinal plants [6].

For preventing amoebiasis, we wash vegetables before consumption, maintain good hygiene, drink clean water, and avoid unhygienically prepared beverages [3, 4].

Here, we present a case combining these two concurrent diseases in 20 days.

## 2. Case Presentation

A 65-year-old, Middle-Eastern male was admitted with a high fever, chills, and vomiting. The symptoms had begun while he was in Liberia, and he came back to Syria as soon as his fever worsened. The patient had no other clinical symptoms and no medical history of other illnesses or drug consumption.

A blood test was performed, and a blood smear was examined under the light microscope (Table 1). He was diagnosed with *Plasmodium falciparum*, and the test was run twice in two different labs. The patient was treated accordingly with artemisinin, 4 tablets per dose twice a day for three days (each tablet contains 20 mg artemether and 120 mg lumefantrine).

Twenty days after malaria treatment, the patient continued to suffer from fever, shivers, and general fatigue. Clinical presentation included pale skin, mucosa, and right hypochondriac pain without jaundice. A blood test with further lab examinations was performed (Table 2).

Blood smears were run for three consecutive days to detect *Plasmodium falciparum* organisms, while a computed tomography (CT) scan and abdominal echography were also scheduled.

**Figure 1:**
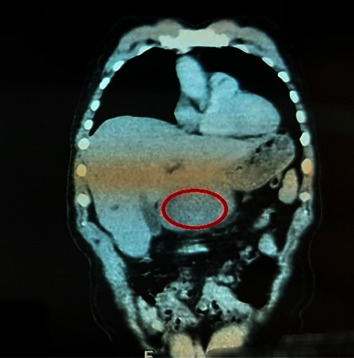
Reformatted coronal contrast-enhanced CT demonstrates a large cystic lesion with a relatively thin wall in the hepatic segment IVb. CT findings are compatible with hepatic abscess.

**Figure 2:**
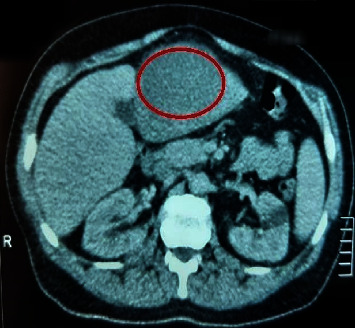
Axial contrast-enhanced CT demonstrates a large cystic lesion with a relatively thin wall in the hepatic segment IVb. CT findings are compatible with hepatic abscess.

The smears returned negative, and the CT scan revealed liver abscesses (Figures 1 and 2); thus, further tests were required.

A blood test was performed searching for *Echinococcus granulosus* antibodies and *Entamoeba histolytica* antibodies, and the test rendered positive results for *Entamoeba histolytica.*

The abscess was drained as a therapeutic and diagnostic measure, followed by a course of 500 mg intravenous metronidazole every 8 hours for 14 days (Flagyl), to complete the treatment. After improvement, the patient was discharged and instructed to contact his physician if any of his symptoms relapsed, which did not occur.

## 3. Clinical Discussion

Malaria is an endemic infection in Liberia, making it one of the most important differential diagnoses for our patient's symptoms on his first visit, meaning it should be ruled out first, especially with the presence of thrombocytopenia [7, 8]. Other differential diagnoses included dengue fever, enteric fever, rickettsial diseases, and HIV-associated infections [8]. The nonspecific nature of the patient's symptoms, his unremarkable physical examination, and the blood smear led the medical team to diagnose uncomplicated malaria [1]. Artemisinin-based combination therapy is the drug of choice for uncomplicated malaria, especially with the global spread of chloroquine-resistant strains [1]. Despite the growing concerns about artemisinin resistance, it remains highly effective in Liberian strains [9]. At the patient's second admission, in addition to the previously mentioned symptoms, the patient suffered from right hypochondriac pain. These symptoms could not be explained by the malaria diagnosis as the blood smears returned negative on three consecutive days. After further investigation, an amoebic liver abscess was diagnosed. The timeline of this infection suggests that the patient contracted the parasite in Liberia. The continuity of the patient's symptoms may suggest that the abscess was already present during the patient's first visit. In other words, one train can hide another: the diagnosis of malaria overshadowed the symptoms and signs of the amoebic abscess.

Three species of intestinal amoeba exist. *E. histolytica* is most frequently symptomatic, *Entamoeba dispar* is largely considered nonpathogenic, and *Entamoeba moshkovskii* has unclear pathogenicity. *E. histolytica's* invasiveness arises from its proton and tissue lysing properties [3]. Risk factors for developing amoebic liver abscesses include male sex, indigenously brewed alcoholic beverages consumption, young age, poor nutrition, immunosuppression, and corticosteroids [3, 4].

Albeit coinfection of *P. falciparum* and *E. histolytica* was previously recorded, predominantly in Sub-Saharan Africa [10]. It remains uncertain whether a connection between the two infections exists. One possible connection in our case would be through the disturbance in ferritin levels. Malaria could have caused elevated ferritin concentrations regardless of its severity, which may have made the patient's liver vulnerable to *Entamoeba histolytica*, which uses iron in ferritin to grow and multiply [11, 12].

Another connection might be through *Plasmodium falciparum's* role in causing the failure of dendritic cells' function [13]. Liver dendritic cells respond tolerantly to *Plasmodium falciparum* parasites, which prevent harmful immunopathology and suppress inflammatory responses. One study even suggests that the low levels of plasmodium can downregulate signals needed for antigen-presenting cell (APC) activation [14]. On the other hand*, Entamoeba histolytica* has a surface molecule named lipopeptidophosphoglycan (LPPG) which binds to TLR2 and TLR4 on the surface of dendritic cells. This shows that LPPG activates APC and therefore is essential in regulating the inflammatory response in amoebiasis [15]. This might mean that the malaria infection predisposed the patient to the amoebic abscess through immunosuppression in the liver.

No further tests were run after the malaria infection was confirmed. However, had the admitting doctor screened for other infectious diseases endemic to Liberia, the amoebic infection could have been noticed earlier, leading to a better treatment plan and prognosis, especially with *E. histolytica* being the third most detected intestinal pathogen in travelers returning from low-income countries presenting with infectious gastrointestinal disease [4].

Here, we stress the importance of a complete examination, and we suggest a cost-benefit study to decide whether symptomatic returning travelers should be screened for the most prevalent endemic infections even if a diagnosis was reached.

## 4. Conclusion

Physical examination should always be thorough, and all signs and symptoms should be accounted for even if a lab diagnosis was made. While treating a returning traveler suffering from an infectious disease, it is imperative to check for other infectious diseases endemic to the area, especially for travelers returning from Sub-Saharan Africa, where coinfections are more common.

Furthermore, we recommend a wait-and-see approach besides precise examinations as the second infection might manifest itself after treatment of the first.

Finally, we propose that the relationship between malaria and amoebic liver abscesses be explored by researching the effects of malaria on ferritin levels and the immune components in the liver and whether it makes the host susceptible to amoebic liver abscesses.

## Figures and Tables

**Table 1:** Blood sample lab results at the first admission show significantly elevated CRP levels and slightly elevated ESR compatible with an acute malaria infection.

White blood cells: normal 4,500 to 10,000 cells/mm^3^ (^∗^10^3^/mm^3^)	Neutrophil (%): normal 40% to 60%	HB (g/dl): normal 13 or higher	Platelets: (^∗^1000/mm^3^)	Creatinine (mg/dl)	Erythrocyte sedimentation rate: normal male: ≤15 mm/hr	C-reactive protein (up to 5 mg/L)
4100	80	12.9	68	1.9	20	188

**Table 2:** Blood sample lab results at the second admission show significantly elevated CRP, ESR, and ferritin levels.

White blood cells: normal 4,500 to 10,000 cells/mm^3^	Neutrophil (%): normal 40% to 60%	Hemoglobin (g/dl): normal 13 or higher	Mean corpuscular volume: 89.8–93.6 fl	Platelets: 150–450 (^*∗*^1000/mm^3^)	Erythrocyte sedimentation rate: normal male: ≤15 mm/hr	C-reactive protein (up to 5 mg/L)	Reticulocytes (%): around 0.5% to 2.5%	Total bilirubin (up to 1 mg/dl)	Direct bilirubin (up to 0.2 mg/dl)	Iron (60–160 *μ*g/dl)	Total iron-binding capacity (250–450 *μ*g/dl)	Ferritin (24–336 ng/ml)	LDH, lactate dehydrogenase (210–480 U/L)
17800	75	6.9	86.4	603	115	286.59	1.5	0.59	0.2	52	260	1847	323

## Data Availability

No data were used to support the findings of this study.

## Abbreviations

APC: Antigen-presenting cells
LPPG: Lipopeptidophosphoglycan
CT: Computed tomography.
